# Discharged

**Published:** 1888-10-20

**Authors:** 


### DISCHARGED.

(The Convalescent's Drive Home.)

Carry me out
Into the wind and the sunshine,
Into the beautiful world.

O the wonder, the spell of the streets!
The stature and strength of the horses,
The rustle and echo of footfalls,
The flat roar and rattle of wheels!
A swift tram floats huge on us......
It's a dream!
The smell of the mud in my nostrils
Is brave—like a breath of the sea!

As of old,
Ambulant, undulant drapery,
Vaguely and strangely provocative,
Flutters and beckons. O yonder—
Scarlet!—the glint of a stocking!
Sudden a spire,
Wedged in the mist! O the houses,
The long lines of lofty, gray houses;
Cross-hatched with shadow and light,
These are the streets......
Each is an avenue leading
Whither I will!

Free...... !
Mizzy, hysterical, faint,
I sit, and the carriage rolls on with me
Into the wonderful world.

